# Hospital Costs of Foreign Non-Resident Patients: A Comparative Analysis in Catalonia, Spain

**DOI:** 10.3390/ijerph14091062

**Published:** 2017-09-14

**Authors:** Elena Arroyo-Borrell, Gemma Renart-Vicens, Marc Saez, Marc Carreras

**Affiliations:** 1 Health Policy Research Unit (SEPPS), Consortium of Health and Social of Catalonia, 08022 Barcelona, Spain; elena.arroyo@udg.edu; 2 Research Group on Statistics, Econometrics and Health (GRECS), University of Girona, Carrer de la Universitat de Girona 10, Campus Montilivi, 17003 Girona, Spain; marc.saez@udg.edu; 3 CIBER of Epidemiology and Public Health (CIBERESP), 28029 Madrid, Spain; 4 GRESSiRES, Research Group on Health Services and Health Outcomes, Serveis de Salut Integrats Baix Empordà, 17230 Palamós, Spain; mcarreras@ssibe.cat; 5 Department of Business Studies, University of Girona, 17004 Girona, Spain

**Keywords:** patient mobility, healthcare costs, foreign non-resident patients, regional public hospitals

## Abstract

Although patient mobility has increased over the world, in Europe there is a lack of empirical studies. The aim of the study was to compare foreign non-resident patients versus domestic patients for the particular Catalan case, focusing on patient characteristics, hospitalisation costs and differences in costs depending on the typology of the hospital they are treated. We used data from the 2012 Minimum Basic Data Set-Acute Care hospitals (CMBD-HA) in Catalonia. We matched two case-control groups: first, foreign non-resident patients versus domestic patients and, second, foreign non-resident patients treated by Regional Public Hospitals versus other type of hospitals. Hospitalisation costs were modelled using a GLM Gamma with a log-link. Our results show that foreign non-resident patients were significantly less costly than domestic patients (12% cheaper). Our findings also suggested differences in the characteristics of foreign non-resident patients using Regional Public Hospitals or other kinds of hospitals although we did not observe significant differences in the healthcare costs. Nevertheless, women, 15–24 and 35–44 years old patients and the days of stay were less costly in Regional Public Hospitals. In general, acute hospitalizations of foreign non-resident patients while they are on holiday cost substantially less than domestic patients. The typology of hospital is not found to be a relevant factor influencing costs.

## 1. Introduction

In the recent years, patient mobility has increased over the world thanks to globalization fostering the movement of people, goods and ideas [1,2]. Furthermore, not only are the numbers of people moving around the world who need some kind of medical care increasing [3], but so too are those who travel abroad to receive treatment in a country where they are non-residents (so-called medical tourists) [4]. The European public health is facing a new challenge: healthcare systems that must be shared by local residents and foreign patients with all the consequences this may have in terms of costs and uses. During the last decade, the patient mobility debate appeared on the European health policy agenda and some recommendations have already been made [5]. However, although there is a lot of literature concerning this phenomenon [2], one finds few empirical studies in Europe tackling patients’ mobility. Due to the lack of comparable and detailed data, little is known about its benefits and challenges [2,6,7]. This is vital research to be able to fund and properly plan the medical needs of foreign patients in the destination country [6,8]. 

Some studies from the United States (U.S.) already highlighted interesting differences between foreign and domestic patients, i.e., the length of hospital stay (LOS) is associated with the patient’s geographic origin [9,10]. Unfortunately, in Europe there are not these kinds of analyses in spite of the increasing mobility of patients. With the exception of some specific subjects, e.g., the analysis of healthcare resources required by economic immigrants [11], the literature related to patients mobile throughout Europe is still in its infancy [6]. Rosenmöller et al. [2] propose five categories of mobile patients in Europe: (1) citizens on holidays, (2) pensioners or retired long-term residents, (3) cross-border care, (4) medical travellers and (5) people sent abroad by their national healthcare system [3,4]. In our case, the concept “foreign non-resident patient” we used throughout the article, mostly coincides with the first category of mobile patients as described by Rosenmöller et al. [2]: foreign people who require healthcare assistance while they are on holiday in Catalonia (Spain). 

In the Catalan Health System 3740 foreign non-residents were hospitalized in 2003, 5627 in 2006 [12] and 4764 in 2012 [13]. Given the significant amount of foreign non-resident hospitalisations, some questions arise: (1) Are there any differences in terms of characteristics between the domestic patient and the foreign non-resident patient? (2) Is it more expensive to treat an international patient? And (3) How international patients are attended within the Catalan hospital network? The aim of this article was three-fold: first, to compare characteristics of foreign non-resident patients versus domestic patients for the particular Catalan case; second, to compare the costs and the utilization of medical resources of foreign non-resident patients versus domestic patients; and, third, to investigate differences between the costs of the foreign non-resident patients depending on the typology of the hospital they are treated.

Information on hospitalization episodes of foreign non-resident patients in tourist areas is important for healthcare providers’ capacity to adjust to the expected demand for their services. Moreover, a better allocation of medical resources would benefit the quality of services for both the foreign non-resident and domestic patient alike. On the other hand, information on the costs of acute hospitalization of foreign non-resident individuals on holiday may help healthcare financing authorities negotiate fair international agreements with the countries of the holidaying citizen’s origin.

## 2. Materials and Methods

### 2.1. Data Setting

This paper used data from two datasets. First, from the 2012 Minimum Basic Data Set-Acute Care (though the abbreviation in English is MBDS-HA, here CMBD-HA will be used) of Serveis de Salut Integrats Baix Empordà (SSIBE) (Catalonia), for both resident and foreign non-resident patients provided by the SSIBE. These data included information about 197 foreign non-resident patients and 9154 domestic patients. SSIBE is an integrated health care network (IHN) and the management organisation is responsible for the provision of public health services, including primary care, specialised attention and acute hospitalisations in the Baix Empordà region in Catalonia (Spain). As the entire data provided by SSIBE concerned acute hospitalisations, hereinafter we will refer to SSIBE as the Palamós Regional Public Hospital. Second, we also used data from the 2012 CMBD-HA hospitals in Catalonia for foreign non-resident patients. In this case, the 2012 CMBD-HA was provided by the Catalan Government’s Department of Health and included information on 4764 foreign non-resident patients. The CMBD in Catalonia began in 1990 as a systematic register of morbidity and healthcare activity. It was made mandatory for all public and private hospitals in Catalonia and it provides a useful record of pathologies treated. The CMBD-HA registers data on the care activity of acute-care hospitals, including standard hospitalization, aimed at patients with some medical or surgical pathology that need continued care, major ambulatory surgery, which does not required the patient’s admission, and minor ambulatory surgery, which includes a simple surgical procedure. 

In this paper, consistent with our objective, we used both samples to describe foreign non-resident patient characteristics and to capture the differences depending on whether the patient is a foreign non-resident or a domestic patient treated at the same hospital and on where the foreign non-resident patient has been treated. We identified two case-control studies: first, the foreign non-resident patients treated at the Palamós Regional Public Hospital were defined as cases, and the domestic patients treated in this same hospital were controls; second, the cases were the foreign non-resident patients treated in Regional Public Hospitals and the controls the foreign non-resident patients treated in other types of hospitals.

To create comparable analysis, in each case-control study we matched the case-control groups on the basis of the following variables: sex (male or female), age, type of activity (major ambulatory surgery or standard hospitalization), LOS (in days), financial system (e.g., free insurance, CatSalut public insurance, out-of-pocket) and admission circumstances (urgent or scheduled). Each case could be matched to only one control. After matching, for the first case-control study, in which we compared foreign non-resident to domestic patients both treated at the Palamós Regional Public Hospital, we had 197 observations. For the second case-control study, in which we compared foreign non-resident patients treated in Regional Public Hospitals to those treated at any other type of hospital, we had 1215 observations.

The patient demographic characteristics included sex (male, reference group, or female) and age group (≤1, 2–14, 15–24, 25–34, 35–44, 45–54, 55–64, 65–74, reference group, 75–84, ≥85 years). Other variables of our model included LOS, type of hospital in terms of the technological level of the hospital and whether it is a public or private hospital (high-technology monographic private hospitals, high-technology public general hospitals, reference private hospitals with high resolution, reference public hospitals with high resolution, etc.), type of activity (major ambulatory surgery or standard hospitalization) and Diagnosis-related group (DRG) relative weight (the relative cost per unit to treat the DRG related to the average amount of costs, per each case-control study). Throughout the article, we used the AP-DRG 27 version of DRG (3M Health Information System, Wallingford, Connecticut) [14,15].

### 2.2. Episode Costs

Healthcare costs related to hospitalization episodes at the Palamós Regional Public Hospital were individually calculated based on the LOS, minutes of surgery, dialysis sessions, inpatient medication, prosthesis, blood transfusions, laboratory tests, anatomical pathology tests, radiology and other tests carried out between the admission date and the discharge date. The Palamós Regional Public Hospital’s costing system meets with the methodology and principles recommended by the working group from the Spanish Network of Hospital Costs (RECH) [16,17]. Healthcare costs related to hospitalization episodes attended to by other hospitals (CMBD data) were obtained from the RECH cost episode database. Thus, depending on the hospital size, each hospitalization episode was valuated with the average cost from the DRG based on the RECH standard. For 2012, the RECH database included over 220,000 episodes of hospitalisation from 13 different Spanish hospitals [17,18]. 

### 2.3. Cost Modelling

We assumed that the cost was distributed as a gamma, with the following density,

(1) $$\mathrm{Pr}\left(y\right)=\frac{1}{\Gamma \left(s\phi \right)}{\left(\frac{s\phi }{\mu }\right)}^{s\phi }y^{s\phi -1}\exp\left(-s\phi \frac{y}{\mu }\right)$$

where E(*y*) = μ; *φ* was the precision parameter (1/*φ*) was the dispersion, equal to the Var(*y*); *s* > 0 was a fixed scaling; and Г was the gamma function.

In order to estimate the cost (per individual), we specified a generalized linear model (GLM) with a log-link.

(2) $$E\left(Y_{ij}\right)=\mu_{ij}\;\mu_{ij}=\exp\left(\eta_{ij}\right)$$

where the subscript *i* denoted the case-control group (match = 1 and control = 0) and *j* denoted each case-control matched group (*j* = 1~197 in the 1st case/control & 1~1215 in the 2nd case/control); and *η* the linear predictor.

In the additive linear predictor *η* in (2) we introduced the variables that would be able to explain the cost:

(3) $$\begin{array}{cc} \eta_{ij}= & \beta_{0j}+\beta_{1j}case\_control_{ij}+\beta_{2}Sex_{ij}+\sum_{k=3}^{11}\beta_{k}Age\_group_{kij}+\\ & \beta_{12}LOS_{ij}+\sum_{k=13}^{22}\beta_{k}Hospital\_level_{kij}+\beta_{23}Activity\_type_{ij}+\beta_{24}GRD\_weight_{ij} \end{array}$$

Note that some of the coefficients had subscripts that indicated the number of parameters. We specified random coefficient panel data models. In mixed models terminology, we allowed (some of the) coefficients to be random effects [19], i.e., to be different for the various levels we considered. Thus, we allowed the intercept and the case-control indicator to be different for each case-control group, capturing characteristic individual specifics not already included in the model (i.e., unobserved individual heterogeneity). In this case, we assumed that random effects were identical and independent Gaussian random variables with constant variance. The effect of the DRG weight was also considered a random effect, that is to say, we accepted that the effect may not be linear. As such, we assumed a random walk of order 1 (i.e., independent increments) for the Gaussian random effects vector (although we also assumed a constant variance) [20]. This empirical strategy applied for both case-control studies.

Given the complexity of our model, we preferred to perform inferences using a Bayesian framework. This approach is considered the most suitable to account for model uncertainty, both in the parameters and in the specification of the models ([21], among others). Moreover, only under the Bayesian approach is it possible to model extra variability (not captured by the Gamma link), with relatively sparse data in some cases. Finally, within the Bayesian approach, specifying a hierarchical structure on the (observable) data and (unobservable) parameters, which were all considered as random quantities, is straightforward. In particular, we followed the Integrated Nested Laplace Approximation (INLA) approach [22], within a (pure) Bayesian framework. We used penalising complexity (PC) priors. These priors are invariant to reparameterisations and have robustness properties [23]. All analyses were carried out with the free software R (version 3.2.2) (R Core Team, Vienna, Austria) [24], through the INLA library [21,25].

Previous studies on healthcare cost modelling stated that no single model guarantees a robust estimator for all types of datasets [26,27,28,29]. According the Bayesian framework described above, the choice of the GLM-log Gamma specification was supported by WAIC (Watanabe-Akaike information criteria) and DIC (Deviance information criteria) statistics obtained from preliminary tests.

## 3. Results

### 3.1. Characteristics of the Samples

Summary statistics for each dataset are shown in Table 1. In the total sample of 9351 patients attended to by the Palamós Regional Public Hospital, 2.1% were foreign non-residents patients and 97.9% were domestic patients. Among the foreign non-resident patients, 45.7% were males and the greater proportions of age groups were found in the 2 to 14 and 55 to 84 years old groups. The most common country of residence was France (26.9%), followed by the United Kingdom (15.7%) and the Netherlands (15.2%). The major financing system was Catsalut/International agreements (62.4%), followed by free insurances (23.9%) and out-of-pocket payments (12.7%). Note that the most of these visits were emergency contacts (94.9%) and the mean of the LOS was 5.3 days (SD = 3.8). In the domestic patient dataset, 44.2% were males and the most common ages were from 25 up to 84 years old. Almost all of the visits were financed by Catsalut (97.2%) and 50.4% admissions circumstances were emergency contacts while 49.6% scheduled contacts. Lastly, the LOS mean was 4.9 days (SD = 6.3).

In the total sample of 4764 patients from the CMBD dataset, 25.5% were those patients attended to by Regional Public Hospitals and 74.5% attended to by any other type of hospital in Catalonia. In both cases, greater rates of men hospitalizations were found (53% and 57.1%, respectively) and the most common ages were from 35 to 84 years old. We could find some differences in terms of residence countries. In the first group, France (32.3%) was the main country of residence of the patients followed by Germany (14.7%) and United Kingdom (12.1%). In the second group, Andorra (26.2%) was the main country, followed by Italy (10.1%), the United Arab Emirates (7%) and France (6.7%). Also, some differences could be found in terms of financing systems. The main financing systems for patients going to Regional Public Hospitals were free insurances (40.6%) and Catsalut/International agreements (39.4%), followed by the out-of-packet payments (16.3%). By contrast, in patients treated at other type of hospitals, 32.8% were out-of-pocket payments, 30% free insurances, and finally 20.2% Catsalut/International agreements. It was worth noting that, there were also differences in admission circumstances: in patients treated in Regional Public Hospitals, 92.8% were emergency contacts whereas this was only a 40.7% in those patients treated in other type of hospitals. Finally, LOS mean of foreign non-resident patients using Regional Public Hospitals was 4.74 days (SD = 4.2) and for other types of hospital was 5.31 days (SD = 6.5).

The ten most frequent DRGs for each group are shown in Table 2. For the first case-control study, the DRGs *5*41. Simple Pneumonia and Oth Respiratory Disord Exc Bronchitis, Asthma and 544. Chf and Cardiac Arrhythmia W Major CC were among the most frequent for both cases and controls. Looking at the second case-control study, only the DRG 541. Simple Pneumonia and Oth Respiratory Disord Exc Bronchitis, Asthma remained among the most frequent for both analyses. Not surprisingly, six of the most frequent DRGs (541, 544, 236, 816, 243, 219) for cases in the first case-control study were at the same time among the most frequent for cases in the second (both are the same typology of hospital). This fact revealed that the nature of acute episodes of activity for foreign non-resident patients was quite similar throughout the two data sets we analysed.

### 3.2. First Case-Control Study

The first case-control study attempted to identify the healthcare costs differences between domestic (control) and foreign non-resident (cases) patients treated at the Palamós Regional Public Hospital. The aim was to assess whether there were differences in healthcare costs depending on the residence country of the patients who were treated in the same hospital. The results of Table 3 show that the healthcare cost of the foreign non-resident patients treated at the Palamós Regional Public Hospital was 12% lower than resident patients (95% credibility interval 2–20%), after adjusting for sex, age, type of activity and LOS. The results of the interactions showed that, in particular, foreign non-resident patients under 14 years old had 27% higher costs than resident patients (*p* < 0.1). However, although the healthcare costs of non-resident patients were not significantly different between men and women, we observed differences in the specific age group from 2 to 14 years old and, in this case, the healthcare costs for women were 19% higher (*p* < 0.1). 

### 3.3. Second Case-Control Study

The second case-control study tried to estimate healthcare cost differences between foreign non-resident patients treated at Regional Public Hospitals (cases) and the foreign non-resident patients treated at other types of hospitals (controls) (Table 4). The aim of this case-control analysis was to assess whether the healthcare system was used efficiently, as similar cases but treated in different types of hospitals should have similar costs if the use is efficient. The results obtained revealed non-significant differences between the healthcare cost of foreign non-resident patients treated at Regional Public Hospitals and the foreign non-resident patients treated in other types of hospitals, after adjusting for sex, age, type of activity, LOS, financing system and the hospital typology (Table 4). However, due to the wide credibility interval of the case-control coefficient, this result must be interpreted with caution. Using the interactions we observed significant differences in some specific subgroups. First, costs of foreign non-resident women treated at Regional Public Hospitals were 2% lower (95% credibility interval 1–4%). Patients treated in Regional Public Hospitals aged 2–14 years old had higher healthcare costs (95% credibility interval 6–18%), whereas those aged between 15–24 and 35–44 had lower healthcare costs (95% credibility interval 1–11% and 1–12%, respectively). We also observed that patients under 1 year old treated in Regional Public Hospitals had higher costs (*p* < 0.1), whereas the costs were lower for patients aged 75–84 years old. Finally, women aged 25–34 years old had, in general, lower costs, whereas women from 55–64 years old had higher costs (*p* < 0.1) (comparing Regional Public Hospitals to other typologies). The interaction between LOS and the case-control group showed that for each day of hospitalisation the costs increased, with the exception of the Regional Public Hospitals, where the costs were reduced by 1% for each day of stay. It is important to highlight that we did not observe significant differences in the financing systems used by the foreign non-resident patient. 

## 4. Discussion

This work represents a first approach to the study of a comparison between domestic and foreign non-resident patients in Catalonia, focusing on patient characteristics, hospitalization costs and differences in costs depending on the typology of the hospital they are treated. The results show differences between domestic and foreign non-resident patients’ characteristics, and between foreign non-residents patients’ characteristics depending on the hospital typology they are treated. The results of the first case-control study show that healthcare costs of foreign non-resident patients are lower than the healthcare costs of domestic patients, both treated in the Regional Public Hospital of Palamós. The second case-control study show that there are not differences between healthcare costs of foreign non-resident patients treated in different kinds of hospitals. This analysis determines that patients are treated efficiently in the healthcare system of Catalonia. 

As we have stated, we presume that the foreign non-resident patients in our samples are patients on holidays (especially patients attended to by Regional Public Hospitals). The characteristics of our samples presented in Table 1 are different from other studies with similar aims using medical traveller patients [9,10]. Apart from having a greater percentage of young patients in our samples, the main differences come from the countries of origin, financing systems, admission circumstances and LOS [13]. Specifically, it is in terms of admission circumstances where we can find larger differences. In the data from Satjapot et al. [9] and Bower et al. [10] most of the admissions are scheduled contacts (77% and 88.5%, respectively). By contrast, in our case rates are much lower, with the exception of the other types of hospital patients (59.3%). Moreover, foreign non-resident patients attended to by Regional Public Hospitals have a high percentage of emergency contacts (94.9% for the first-case control sample, 92.8% for the second). This higher percentage is consistent with the idea of having an unexpected health event while on holiday. Finally, while LOS are longer in the studies of Satjapot et al. [9] and Bower et al. [10] (means of 7 and 6.94 days, respectively), in our case we find, at maximum, 5.31 days of stay in patients attended to by other type of hospitals. 

It can be stated that patients treated in other type of hospitals (second-case control) are more similar to the previous papers. In this case, the large percentage of scheduled contacts, together with the mix of activity (most frequents DRGs) and the high percentage of individuals from the neighbouring countries, suggests that our data set may contain an important number of episodes of cross-border care, as described by Rosenmöller et al. [30]. Despite the lack of comprehensive bilateral agreements on health services cooperation, it is especially remarkable the high percentage of discharges related to individuals from Andorra (26.2%), which are mostly reimbursed by free insurances.

A limitation of our work, as in any Bayesian analysis, is that the choice of the prior distributions of model parameters (i.e., priors) may have had a considerable impact on the results. However, we used priors that penalize the complexity (PC priors) [23] that have been found to be very robust. Furthermore, we performed sensitivity analyses to assess how the prior on the hyperparameters influenced the estimation results. First, by increasing the precision (lowering the variance) and second, by testing other priors, those used by default in R INLA (log gamma) with different shape and inverse-scales; uniform and centered half-normal. PC priors provided better results in all cases. The complete set of preliminary tests are available form the authors under request.

A second limitation of the study concerns external validity. Our results on patient mobility described the particular Catalan case, mostly including foreign people who required health care assistance while they are on holiday. A different healthcare system attending a different flow of mobile patients may come to different results. Regarding internal validity, both case control samples included all foreign non-resident patients attended in the Palamós Regional Public Hospital/Catalonia in 2012, without relevant cost differences attributable to the typology of hospital. Therefore, no selection bias was plausible.

Another important limitation of this paper is the lack of similar studies. Despite the fact that the mobility of patients is increasing in Europe, there are few empirical studies, which do not allow us to compare and discuss our results with other similar work.

Further research is required regarding retired long-term residents, the second category described by Rosenmöller et al. [2,31]. According to official statistics, the percentage of foreign residents in Catalonia in 2012 was 15.68% [32]. Several studies analysed public hospital utilisation by foreign residents in Spain, specially focusing on immigrant population [33,34,35]. In general, previous studies found lower rates of utilisation, which were attributed to age and better health conditions among the immigrant population [11,36,37]. However, given that average utilisation rates by immigrants are not comparable to those by pensioners; more research focussed on specific groups among foreign residents is required. 

## 5. Conclusions

Except for individuals under 14 years old, acute hospitalizations of foreign non-resident patients while they are on holiday cost substantially less than domestic patients. The typology of hospital is not found to be a relevant factor influencing costs.

Concerning cross-border care, healthcare planners should monitor the most prevalent episodes related to individuals living in the border countries and the corresponding financing agreements involved, especially for high level hospitals.

## Figures and Tables

**Table 1:** Summary statistics.

Variable	Patients Attended to by the Palamós Regional Public Hospital (n = 9351)		Patients Attended to by Catalan Hospitals (CMBD) (n = 4764)	
	Non-Resident Patients	Domestic Patients	Regional Public Hospitals	Other Types of Hospitals
	N (%) or Mean (SD)		N (%) or Mean (SD)	
Total ^a^	197 (2.1%)	9154 (97.9%)	1215 (25.5%)	3549 (74.5%)
Sex				
Male	90 (45.7%)	4043 (44.2%)	644 (53%)	2025 (57.1%)
Female	107 (54.3%)	5111 (55.8%)	571 (47%)	1521 (42.9%)
Age (years old)	51.5 (27)	52.6 (25.9)	52 (25.6)	44.3 (23.2)
0–1	6 (3%)	511 (5.6%)	30 (2.5%)	149 (4.2%)
2–14	24 (12.2%)	446 (4.9%)	100 (8.2%)	343 (9.7%)
15–24	12 (6.1%)	390 (4.3%)	107 (8.8%)	313 (8.8%)
25–34	12 (6.1%)	1121 (12.2%)	94 (7.7%)	401 (11.3%)
35–44	17 (8.6%)	970 (10.6%)	110 (9.1%)	439 (12.4%)
45–54	10 (5.1%)	944 (10.3%)	104 (8.6%)	522 (14.7%)
55–64	39 (19.8%)	1019 (11.1%)	180 (14.8%)	561 (15.8%)
65–74	32 (16.2%)	1341 (14.6%)	221 (18.2%)	530 (14.9%)
74–84	32 (16.2%)	1744 (19.1%)	187 (15.4%)	237 (6.7%)
≥85	13 (6.6%)	668 (7.3%)	82 (6.7%)	50 (1.4%)
Major countries residence ^b^				
France	53 (26.9%)		393 (32.3%)	237 (6.7%)
The United Kingdom	31 (15.7%)		147 (12.1%)	199 (5.6%)
The Netherlands	30 (15.2%)		130 (10.7%)	93 (2.6%)
Germany	19 (9.6%)		178 (14.7%)	143 (4%)
Belgium	17 (8.6%)		68 (5.6%)	49 (1.4%)
Switzerland	12 (6.1%)		35 (2.9%)	51 (1.4%)
Russia	6 (3%)		41 (3.4%)	64 (1.8%)
Italy	2 (1%)		41 (3.4%)	360 (10.1%)
Type of activity				
Major ambulatory surgery	4 (2%)	2599 (28.4%)	32 (2.6%)	579 (16.3%)
Standard hospitalization	193 (98%)	6555 (71.6%)	1183 (97.4%)	2970 (83.7%)
Major financial systems ^b^				
Free insurances	47 (23.9%)	62 (0.7%)	493 (40.6%)	1065 (30%)
The individual	25 (12.7%)	5 (0.1%)	198 (16.3%)	1163 (32.8%)
Catsalut/International agreement	123 (62.4%)	0	479 (39.4%)	716 (20.2%)
Catsalut	0	8895 (97.2%)	0	0
Major types of other hospitals				
High-tech general public Hospitals				751 (21.2%)
Reference private hospitals with high resolution				649 (18.3%)
High-tech monographic private hospitals				870 (24.5%)
Reference public hospitals with high resolution				470 (13.2%)
Admission circumstances				
Emergency contact	187 (94.9%)	4613 (50.4%)	1127 (92.8%)	1445 (40.7%)
Scheduled contact	10 (5.1%)	4541 (49.6%)	88 (7.2%)	2104 (59.3%)
Length of stay (LOS)	5.3 (3.8)	4.9 (6.3)	4.74 (4.2)	5.31 (6.5)
Cost	2228.7 (2240.4)	2165.3 (2478.1)	2709.8 (3294)	4313.1 (5519.5)

^a^: The aggregate sum of individuals of the category of each group not necessarily coincides with the total sum due to the existence of data missing at random. ^b^: The major cases taking into account the Regional Public Hospitals. SD: Standard deviation.

**Table 2:** The ten most frequent DRG for each group.

**Patients Attended to by the Palamós Regional Public Hospital**			
**Non-Resident Patients**		**Domestic Patients**	
**DRG**	**Frequency (%)**	**DRG**	**Frequency (%)**
541. Simple pneumonia and oth respiratory disord exc bronchitis, asthma	8 (4.1%)	039. Lens procedures with or without vitrectomy	790 (8.6%)
381. Abortion W D and C, aspiration curettage or hysterotomy	6 (3%)	373. Vaginal delivery W/O complicating diagnoses	540 (5.9%)
167. Appendectomy W/O complicated principal diag W/O CC	6 (3%)	541. Simple pneumonia and oth respiratory disord exc bronchitis, asthma	267 (2.9%)
544. CHF and cardiac arrhythmia W major CC	6 (3%)	372. Vaginal delivery W complicating diagnoses	248 (2.7%)
236. Fractures of hip and pelvis	5 (2.5%)	006. Carpal tunnel release	220 (2.4%)
816. Nonbacterial gastroenteritis and abdominal pain age < 18 W/O CC	5 (2.5%)	629. Neonate, bwt > 2499 g, W/O signif O.R. proc, W normal newborn diag	200 (2.2%)
455. Other injury, poisoning and toxic effect diagnosis W/O CC	5 (2.5%)	162. Inguinal and femoral hernia procedures age > 17 W/O CC	199 (2.2%)
243. Medical back problems	5 (2.5%)	225. Foot procedures	178 (1.9%)
219. Lower extrem and humer proc exc hip, foot, femur age > 17 W/O CC	5 (2.5%)	359. Uterine and adnexa proc for Ca in situ and non-malignancy W/O CC	170 (1.9%)
252. Fx, Sprn, Strn and Disl of forearm, hand, foot age < 18	4 (2%)	544. CHF and cardiac arrhythmia W major CC	161 (1.8%)
**Patients Attended to by Catalan Hospitals (CMBD)**			
**Regional Public Hospitals**		**Other Type of Hospitals**	
**DRG**	**Frequency (%)**	**DRG**	**Frequency (%)**
541. Simple pneumonia and oth respiratory disord exc bronchitis, asthma	46 (3.8%)	039. Lens procedures with or without vitrectomy	362 (10.2%)
243. Medical back problems	31 (2.6%)	042. Intraocular procedures except retina, iris and lens	224 (6.3%)
373. Vaginal delivery W/O complicating diagnoses	30 (2.5%)	040. Extraocular procedures except orbit age > 17	135 (3.8%)
219. Lower extrem and humer proc exc hip, foot, femur age > 17 W/O CC	27 (2.2%)	036. Retinal procedures	83 (2.3%)
167. Appendectomy W/O complicated principal diag W/O CC	26 (2.1%)	041. Extraocular procedures except orbit age < 18	71 (2%)
816. Nonbacterial gastroenteritis and abdominal pain age < 18 W/O CC	26 (2.1%)	758. Back and neck procedures except spinal fusion W/O CC	64 (1.8%)
236. Fractures of hip and pelvis	25 (2.1%)	756. Spinal fusion W/O CC	46 (1.3%)
127. Heart failure and shock	25 (2.1%)	883. Laparoscopic appendectomy	41 (1.2%)
544. CHF and cardiac arrhythmia W major CC	24 (2%)	143. Chest pain	38 (1.1%)
211. Hip and femur procedures except major joint age > 17 W/O CC	21 (1.7%)	541. Simple pneumonia and oth respiratory disord exc bronchitis, asthma	37 (1%)

DRG: Diagnosis-related group; D: Dilation; C: Curettage; W: With; W/O: Without; CC: Complications.

**Table 3:** First case-control study (non-resident vs. resident patients treated at the Palamós Regional Public Hospital).

Main Coefficients	RR (95% CI)
Intercept	939.64 (767.68–1149.30) **
Case control	0.88 (0.80–0.98) **
Interactions	
Case control * age (≤1)	1.28 (0.90–1.81) *
Case control * age (2–14)	1.27 (0.94–1.71) *
Case control * age (15–24)	1.11 (0.84–1.47)
Case control * age (25–34)	0.97 (0.67–1.40)
Case control * age (35–44)	0.90 (0.59–1.39)
Case control * age (45–54)	0.98 (0.66–1.44)
Case control * age (55–64)	0.97 (0.70–1.34)
Case control * age (75–84)	0.83 (0.61–1.13)
Case control * age (≥85)	0.90 (0.66–1.23)
age (≥1) * sex	0.91 (0.66–1.25)
age (2–14) * sex	1.19 (0.92–1.55) *
age (15–24) * sex	0.88 (0.69–1.13)
age (25–34) * sex	1.08 (0.78–1.51)
age (35–44) * sex	0.99 (0.68–1.45)
age (45–54) * sex	1.03 (0.73–1.44)
age (55–64) * sex	1.03 (0.78–1.35)
age (75–84) * sex	0.94 (0.74–1.21)
age (≥85) * sex	0.87 (0.67–1.13)

RR: relative risk; 95% CI: 95% confidence interval. ** *p* < 0.05, * *p* < 0.1. The model is adjusted for sex, age, type of activity and LOS.

**Table 4:** Second case-control study (non-residents patients treated at Regional Public Hospitals vs. other types of hospitals).

	RR (95% CI)
Intercept	235.36 (6.8 × 10^−3^–8.06 × 10^6^)
Case control	8.63 (2.02 × 10^−6^–3.64 × 10^7^)
Interactions	
Case control * sex	0.98 (0.96–0.99) **
Case control * age (≤1)	1.04 (0.99–1.11) *
Case control * age (2–14)	1.12 (1.06–1.18) **
Case control * age (15–24)	0.94 (0.89–0.99) **
Case control * age (25–34)	0.98 (0.93–1.04)
Case control * age (35–44)	0.94 (0.88–0.99) **
Case control * age (45–54)	1.03 (0.98–1.08)
Case control * age (55–64)	0.98 (0.93–1.03)
Case control * age (75–84)	0.97 (0.93–1.01) *
Case control * age (≥85)	0.99 (0.95–1.04)
age (≥1) * sex	1.00 (0.95–1.06)
age (2–14) * sex	1.01 (0.96–1.07)
age (15–24) * sex	0.99 (0.95–1.05)
age (25–34) * sex	0.96 (0.91–1.01) *
age (35–44) * sex	0.99 (0.93–1.05)
age (45–54) * sex	1.02 (0.96–1.07)
age (55–64) * sex	1.04 (0.99–1.09) *
age (75–84) * sex	0.98 (0.93–1.03)
age (≥85) * sex	0.99 (0.95–1.05)
Case control * LOS	0.99 (0.99–0.99) **

** *p* < 0.05, * *p* < 0.1. The model is adjusted for sex, age, length of stay (LOS), type of activity, financial system and type of hospital. RR: relative risk; 95% CI: 95% confidence interval.

## Availability of Data and Materials

The data that support the findings of this study are available from SSIBE and Catalan Government’s Department of Health but restrictions apply to the availability of these data, which were used under license for the current study, and so are not publicly available. Data are however available from the authors upon reasonable request and with permission of SSIBE and Catalan Government’s Department of Health.

## Abbreviations

CMBD-HA	Minimum Basic Data Set-Acute Care
DRG	Diagnosis-related group
GLM	generalized linear model
INLA	Integrated Nested Laplace Approximation
LOS	Length of hospital stay
PC	penalising complexity priors
RECH	Spanish Network of Hospital Costs
SD	Standard deviation
SSIBE	Serveis de Salut Integrats Baix Empordà
U.S.	United States
